# Relationship between total homocysteine, total cholesterol and creatinine levels in overt hypothyroid patients

**DOI:** 10.1186/2193-1801-2-423

**Published:** 2013-08-30

**Authors:** Saleh A Bamashmoos, Mohammed AK Al-Nuzaily, Ali M Al-Meeri, Faisal HH Ali

**Affiliations:** Haematology Department, Faculty of Medicine and Health Sciences, Sana’a University, Sana’a, Yemen; Biochemistry Department, Faculty of Medicine and Health Sciences, Sana’a University, Sana’a, Yemen; Haematology Department, Kuwait University Hospital, Faculty of Medicine and Health Sciences, Sana’a University, Sana’a, Yemen; Department of Nutrition and Dietetics, Faculty of medicine and health sciences, Metabolism and Genomic Programme, Universiti Putra Malaysia, Serdang 43400 UPM, Selangor, Malaysia

**Keywords:** Hypothyroidism, Homocysteine, Cardiovascular disease, Atherosclerosis, Cholesterol, Creatinine, Thyroxine, Triiodothyronine, Thyroid stimulating hormone

## Abstract

Hypothyroidism is associated with an increased risk for cardiovascular disease, which can not be fully explained by the atherogenic lipid profile, particularly total cholesterol and LDL-C, and other pathogenic factors may be involved. Plasma total homocysteine (tHcy) is an independent risk factor for cardiovascular disease and accelerated atherosclerosis. The aim of this study was to investigate the serum total homocysteine (tHcy) levels and its relation to total cholesterol, creatinine and thyroid hormones fT3, fT4 and TSH levels in overt hypothyroid patients compared to control subjects. In this study thirty recently diagnosed, non-treated overt hypothyroid patients (f=27, m=3) and twenty normal volunteers control (f=18, m=2) were included and subjected to determination of serum tHcy by enzyme immunoassay (EIA) technique, fT3, fT4 and TSH by Elecsys cobas e 601 analyzer, total cholesterol by enzymatic method and creatinine by kinetic method. The data was statistically analysed by SPSS-10 and p values less than 0.05 were considered significant.Our results showed that there were a significant increase of tHcy, TSH, T.cholesterol and creatinine levels by 113%, 12-folds, 58% and 54%, respectively, and a significant decrease of fT4 and fT3 levels by 49.6% and 56.4% , respectively, in hypothyroid patients than in control group. For tHcy (Mean±SD, 24.45±5.50 μmol/l vs 11.48±3.03 μmol/l, respectively; P < 0.001). tHcy was significantly positively correlated with TSH, creatinine and age and negatively correlated with free thyroxine (fT4) and no significant correlations with fT3 and T.cholesterol. In conclusion, our study confirmed the observation of elevated serum tHcy, T.cholesterol and creatinine in overt hypothyroidism and the presence of an inverse relation between tHcy with fT4 and a positive relation with TSH.

## Introduction

Hypothyroidism is associated with an increased risk for atherosclerotic cardiovascular disease (Ichiki, 2010) and cardiovascular morbidity (Hak et al., 2000) , which is in accordance with autopsy studies showing that the atherosclerotic process is increased in hypothyroidism (Steinberg, 1968) and decreased in hyperthyroidism (Myasnikov et al., 1963). The increased cardiovascular morbidity in hypothyroid patients has been related to elevated levels of cholesterol and low-density lipoprotein cholesterol (LDL-C), which are normalized after thyoid hormones replacement (Yazbeck et al., 2001). Elevated cholesterol and lipoprotein levels may be partly responsible for the high risk of vascular disease associated with hypothyroidism. However, lipid abnormalities in hypothyroid patients do not fully account for the accelerated atherosclerosis and cardiovascular disease, and other pathogenic factors may be involved (Masaki et al., 1992; Mamiya et al., 1989).

Total homocysteine (tHcy) in plasma is an independent risk factor for cardiovascular disease (Saeed et al., 2006; Virdis et al., 2002). It has often been shown to be related to occlusive vascular disease independently of other known risk factors (den Heijer et al., 2005). Hyperhomocysteinemia induces endothelial injury, oxidative stress, smooth muscle hypertrophy and oxidation of LDL-cholesterol. Platelet aggregation, anticoagulant functions of plasma and vascular vasomotor function are altered in the presence of high plasma levels of Hcy (Medina et al., 2001). The plasma level of tHcy is affected by several genetic and acquired factors and is elevated under conditions of vitamin folate and cobalamin deficiencies and in renal failure (de Bree et al., 2001; Schneede et al., 2000). In the present study we investigated the serum total homocysteine (tHcy) levels and its relation to T.cholesterol, creatinine and thyroid hormones fT3, fT4 and TSH levels in recently diagnosed overt hypothyroid patients compared to control subjects.

## Material and methods

### Subjects

This study was conducted in Sana’a, Yemen, from April to May, 2013. It included 50 subjects aged 20 to 55 years. The patient’s group consisted of 30 newly diagnosed, non-treated overt hypothyroid patients (27 females and 3 males) (mean age ±SD, 37.43±6.92; median, 37.0; ranged from 28 to 55; 95% CI, 34.8-40.0 years old) Patients with any heart diseases, renal diseases and bone diseases and treated patients have been excluded. These patients were selected randomly each day from subjects referred to the out-patient’s clinics of endocrinology and general surgery departments (n=10) of Kuwait University Hospital (KUH) and from subjects referred to the specialized medical laboratories, Al-Aulaqi (n=12) and The Med-Lab. (n=8), for ELISA thyroid hormones measurements, the diagnosis of overt hypothyroidism was based on low levels of serum fT4 and/or fT3 and high TSH levels. The control group included 20 subjects (f=18; m=2) (mean age ±SD, 29.75±6.15; median, 29; ranged from 20–45; 95% CI, 26.9-32.6 years old) as normal non-hypothyroid volunteers from the workers and students of KUH. All participants gave their informed consent to participate in this study.

### Sample collection

Non-fasting blood samples (5ml) of venous blood were collected randomly from each of patients and controls. Sample of plain tube was left to clot for 30 minutes then centrifuged at 3500 × g for 5 minutes; the separated serum was divided into several aliquots and stored at −70°C until analysis and estimation of fT3, fT4 and TSH. The remaining serum samples were stored at – 70°C for later analysis for the estimation of total homocysteine, total cholesterol and creatinine levels.

### Biochemical methods

#### Determination of total homocysteine (tHcy)

Serum tHcy concentrations were determined by a commercially available Axis^®^ Homocysteine enzyme immunoassay (EIA) reagent kit supplied by (Axis-Shield, Axis Biochemicals ASA, Distributed by IBL, Hamburg, Germany) and run on Multiscan EX from Labsystem, Finland. The reference values for adult male and females between 5 and 15 μmol/L and among alderly (>60 years) was 5–20 μmol/L.

#### Determination of thyroid hormones

Serum fT3, fT4 and TSH concentrations were determined by electrochemiluminescence immunoassay (ECLIA) technique intended for use on the Elecsys reagent kits supplied by Roche Diagnostics GmbH (Mannheim, Germany) and run on cobas e 601 immunoassay analyzer from Roche Diagnostics Ltd, Switzerland. The reference range for serum fT3 (2.0-4.4 pg/mL), fT4 (0.93-1.7 ng/dL) and TSH (0.27-4.20 mIU/mL).

#### Determination of total cholesterol and creatinine

Serum total cholesterol and creatinine concentrations were estimated using kits supplied by Randox Lab. Ltd. (United Kingdom) and run on RA-50 Chemistry Analyser from Bayer, Ireland. The reference range for serum total cholesterol (up to 5.18 mmol/L), creatinine (female; 44.2-79.5 μmol/L, male: 53.0-97.2 μmol/L).

#### Statistical analyses

All statistical analyses were performed using the Statistical Package for the Social Sciences (SPSS software version 10.0 for Windows, Inc., Chicago, Illions, USA) to indicate the degree of significant between the mean values of the patient groups and the mean values of the corresponding controls. Descriptive data were given as mean ± standard deviation (SD). All tests were two-tailed and p values less than 0.05 were considered statistically significant. Pearson correlation coefficients (r) were calculated to quantify the relationship between folate and other variables.

## Results

### Total homocysteine (tHcy)

**Figure 1:**
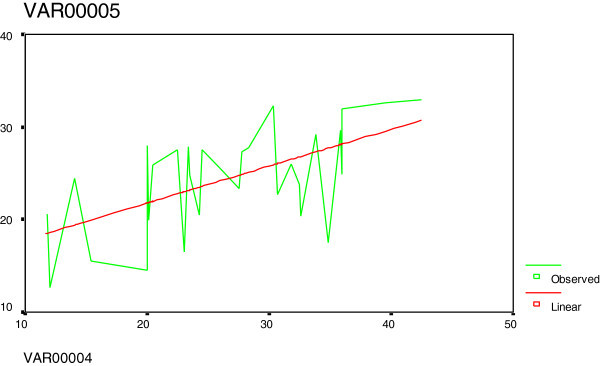
**The relationship between tHcy (var5) and TSH (var4): There was a positive relation.**

**Figure 2:**
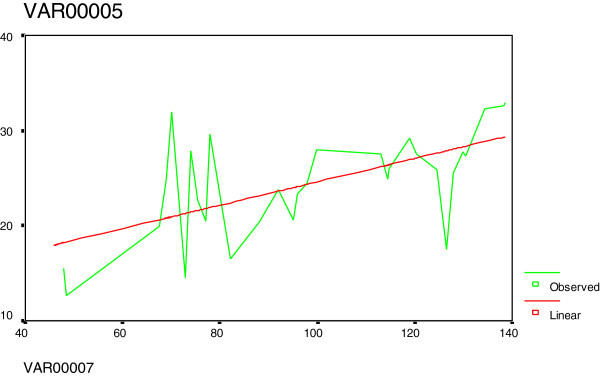
**Curve estimation between tHcy (var5) and creatinine (var7): There was a positive relation.**

**Figure 3:**
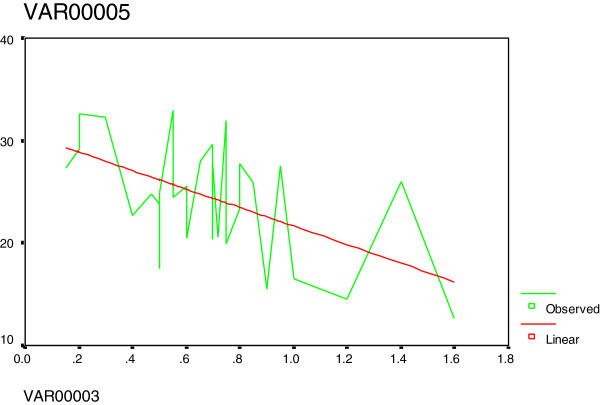
**Curve estimation between tHcy (var5) and fT4 (var3): There was an inverse relation.**

Total homocysteine (tHcy), there was a significant increase mean tHcy in hypothyroid patients than in control group by13.0 μmol/l (113%) (Mean ± SD, 24.45±5.50 μmol/l; 95% confidence interval (CI), 22.40-26.51 vs 11.48±3.03 μmol/l; 95% CI, 10.06-12.90, respectively; p=0.001) (Table 1). The median tHcy of hypothyroid patients was 13.5 μmol/l higher than that of control subjects. In hypothyroid patients, tHcy was ranged from 12.60 to 32.90 as compared to control 6.00 to 18.00 μmol/l. 25 of 30 (83%) hypothyroid patients had tHcy levels >18.0 μmol/L, the upper limit of the control. (Table 2) showed that there were a significant positively correlation in hypothyroid patients between tHcy with TSH levels (p=0.001), creatinine (p < 0.001), and age (p=0.001). In contrast, tHcy was significantly negatively correlated with fT4 (r= −0.535; p=0.002), and non-significantly correlated with fT3 and T.cholesterol (Figures 1, 2 and 3).

**Table 1:** **Comparison between serum levels of tHcy, FT3, FT4, TSH, T.cholesterol and creatinine in hypothyroid patients and control subjects**

Groups	Hypothyroid	Control	P value
Variables	patients (n=30)	subjects (n=20)	
	(Mean ± SD)	(Mean ± SD)	
**Total homocysteine (tHcy) (μmol/L)**	**24.45 ± 5.50**	**11.48 ± 3.03**	**0.001**
**Free triiodothyronine (FT3) (pg/ml)**	**1.46 ± 1.20**	**3.35 ± 0.62**	**0.001**
**Free thyroxine (FT4) (ng/dL)**	**0.69 ± 0.32**	**1.37 ± 0.22**	**0.001**
**Thyroid-stimulating hormone (TSH) (mlU/mL)**	**26.69 ± 8.05**	**2.03 ± 1.09**	**0.001**
**Total cholesterol (mmol/L)**	**6.29 ± 1.56**	**3.98 ± 0.57**	**0.001**
**Creatinine (μmol/L)**	**98.75 ± 27.07**	**64.04 ± 9.53**	**0.001**
**Age (Year)**	**37.43 ± 6.92**	**29.75 ± 6.15**	**0.001**

**Table 2:** **Correlations between serum tHcy and other variables in hypothyroid patients**

Variable	***Hypothyroidism (n=30)***	
	r	P†
**Age (Year)**	**0.557**	**0.001**
**TSH**	**0.582**	**0.001**
**Creatinine**	**0.603**	**0.001**
**FT4**	**−0.535**	**0.002**
**FT3**	**0.204**	**0.279**
**T.cholesterol**	**0.288**	**0.123**

†Pearson correlation, Correlation is significant at the 0.05 level (2-tailed).

### Thyroid hormones parameters

Serum TSH levels in all 30 (100%) hypothyroid patients were significantly higher than the control group by 12.0-folds (Mean±SD, 26.69±8.05 mIU/mL; median, 26.75; ranged from 11.80 to 42.50 vs 2.03±1.09 mIU/mL, median, 2.0; ranged from 0.45 to 4.0, respectively; p=0.001) (Table 1). Serum TSH level was significantly positively correlated with tHcy (r= 0.582; p=0.001) and creatinine (r= 0.482; p=0.007) and negatively correlated with fT4 (r= −0.456; p=0.011) and non-significantly correlated with total cholesterol, fT3 and age. On the other hand, both fT4 and fT3 were observed to be significantly lower in the hypothyroid patients by 49.6% and 56.4%, respectively, compared with the control group (Table 1). For fT4, the mean±SD was 0.69±0.32 ng/dl; median was 0.70; and ranged from 0.15 to 1.60 vs 1.29±0.18 ng/dl, median, 1.35; ranged from 0.97 to 1.80, respectively; p=0.001). Serum fT4 level was significantly negatively correlated with TSH (r= −0.456; p=0.011), tHcy (r= −0.535; p=0.002) and creatinine (r= −0.436; p=0.016) and non-significantly correlated with other variables. For fT3, the mean±SD was 1.46±1.20 pg/ml; median was 0.82; and ranged from 0.25 to 3.83 vs 3.35±0.62 pg/ml, median, 3.45; ranged from 2.20 to 4.20, respectively; p=0.001). Serum fT3 level was non-significantly correlated with other variables. 25 of 30 (83%) hypothyroid patients had fT4 levels < 0.93 ng/dl, the lower limit of assay, whereas 20 of 30 (67%) had fT3 levels < 2.0 pg/ml, the lower limit of assay.

### T.cholesterol and creatinine

Both serum total cholesterol and creatinine levels were significantly higher by 58% and 54%, respectively, compared with the control group (Table 1). For T.cholesterol, the mean±SD was 6.29±1.56 mmol/l; median was 5.97; ranged from 4.20 to 9.90 vs 3.98±0.57 mmol/l; median, 4.18; ranged from 2.80 to 4.80, respectively; p=0.001). For creatinine, the mean±SD was 98.75±27.07 μmol/l; median was 97.0; ranged from 48.0 to 138.5 μmol/l vs 64.0±9.53 μmol/l; median, 65.7; ranged from 45.0 to 77.0, respectively; p=0.001). 23 of 30 (77%) of hypothyroid patients were hypercholesterolemic (>5.18 mmol/l, the upper limit of assay), and 20 of 25 (80%) of hyperhomocysteinemic hypothyroid patients were hypercholesterolemic, whereas 21 of 30 (70%) of hypothyroid patients had elevated serum creatinine levels than the upper limit of assay.

## Discussion

In the present study, elevated serum total homocysteine (tHcy) was found to be strongly associated with hypothyroidism. As shown in (Table 1), serum levels of tHcy were significantly higher in patients with hypothyroidism by 113% compared with the control group (p < 0.001). This observation is in line with other previous studies (Gunduz et al., 2012; Ozmen et al., 2006; Diekman et al., 2001; Lien et al., 2000) and was not in agreement with Orzechowska et al. (2007). They observed that tHcy levels were non-significantly higher in patients with hypothyroidism than in healthy subjects. In our study, tHcy was significantly related to TSH, creatinine and age and negatively related to fT4 and no relations with fT3 and cholesterol. Similar observations have been made in some but not all studies (Orzechowska et al., 2007; Lien et al., 2000; Nedrebo et al., 1998). The increase observed in tHcy in these patients may explain and contribute to a higher cardiovascular risk, since an earlier study indicates that an increase in plasma Hcy level of 4 μmol/l, confers a 40% increase in relative risk for coronary heart disease compared with healthy controls (Boushey et al., 1995).

Increased tHcy levels might be the result of two mechanisms either increased tHcy formation or decreased renal tHcy clearance due to direct effect of thyroid hormones on the tHcy metabolism in the liver and clearance in the kidney (Orzechowska et al., 2005). The former may be explained as thyroid hormone deficiency decreases hepatic levels of enzymes involved in the remethylation pathway of tHcy to methionine, methylenetetrahydrofolate reductase (MTHFR). Experimental studies have also indicated that MTHFR was decreased in hypothyroidism and increased in hyperthyroidism (Selhub, 1999). The later mechanism may be explained as decreased renal tHcy metabolism leads to low renal tHcy, which, in turn, leads to decreased glomerular filtration rate (GFR) and increased serum creatinine (Hollander et al., 2005; Nakahama et al., 2001).

On the other hand, In line with previous studies, we found that serum creatinine was significantly (p < 0.001) higher in hypothyroid patients by 54% compared to the control group. In correlation analysis, there was a positive relation between tHcy and serum creatinine levels in hypothyroid patients. These observations are consistent with other studies (Diekman et al., 2001; Nakahama et al., 2001; Lien et al., 2000). Serum creatinine levels were reported to decrease in hyperthyroidism and increase in hypothyroidism, and elevated levels can be reduced by thyroid hormone replacement (Diekman et al., 2001). The moderate increase of creatinine might be explained by direct effect of thyroid hormones on renal function and/or tHcy metabolism and clearance in kidney (Hollander et al., 2005; Nakahama et al., 2001). The former may be explained by the hypodynamic circulation in hypothyroidism (Polikar et al., 1993). Thyroid hormones are cardiotonic agents, which increase cardiac output while lowering systemic vascular resistance (Klemperer et al., 1995), resulting in increased renal blood flow (Polikar et al., 1993). This, in turn, may increase the glomerular filtration rate (GFR), which is related to serum creatinine (Montenegro et al., 1996), but also closely associated with plasma tHcy (Bostom et al., 1999). The later may be explained as a result of impaired renal tHcy clearance, possibly due to impaired renal tHcy metabolism (Guttormsen et al., 1997). Thus, thyroid hormones may influence the tHcy serum levels both through effects on Hcy formation and its elimination from plasma.

We observed that serum mean total cholesterol levels were significantly higher in patients with hypothyroidism by 58% compared to control group (p < 0.001). These results were consistent with (Gunduz et al., 2012; Yazbeck et al., 2001; Lien et al., 2000). Hypercholesterolemia was seen in 77% of hypothyroid patients, and in 80% of hyperhomocysteinemic hypothyroid patients. Increased serum cholesterol might be attributed to the influence of thyroid hormones on the cholesterol metabolism or disposition (Ness and Lopez, 1995), and this may contribute to the association between hypothyroidism and hypercholesterolemia or it might be attributed to the effects of Hcy on cholesterol production and secretion, since tHcy stimulate the production and secretion of cholesterol in hepatic cells (Karmin et al., 1998) and this may contribute to the association between cholesterol and homocysteine observed in the present study as in some epidemiological studies (Arnesen et al., 1995, Nygard et al., 1995). Since hypercholesterolemia may partly be responsible for increased cardiovascular morbidity, but can not fully explain the accelerated atherosclerosis, the increase in both serum tHcy and cholesterol may confer increased cardiovascular risk observed in these hypothyroid patients.

## Conclusion

In conclusion, our study confirmed the observation of elevated serum tHcy, T.cholesterol and creatinine in overt hypothyroidism and the presence of an inverse relation between tHcy with fT4 and a positive relation with TSH. Increased tHcy levels may contribute to a greater cardiovascular risk. Hyperhomocysteinemia, together with hypercholesterolemia, may explain the accelerated atherosclerosis in these patients and we recommended tHcy screening of hypothyroid patients, as an independent risk factor for accelerated atherosclerosis and cardiovascular disease.
